# Effect of a Physio-Feedback Exercise Intervention Program on the Static Balance of Community-Dwelling Older Adults: A Clustered Randomized Controlled Trial

**DOI:** 10.3390/geriatrics11010006

**Published:** 2026-01-03

**Authors:** Jethro Raphael M. Suarez, Kworweinski Lafontant, Chitra Banarjee, Rui Xie, Joon-Hyuk Park, Ladda Thiamwong

**Affiliations:** 1 Department of Mechanical and Aerospace Engineering, University of Central Florida, Orlando, FL 32816, USA; joon.park@dgist.ac.kr; 2 College of Nursing, University of Central Florida, Orlando, FL 32827, USA; kworweinski.lafontant@ucf.edu (K.L.); rui.xie@ucf.edu (R.X.); ladda.thiamwong@ucf.edu (L.T.); 3 Institute of Exercise Physiology and Rehabilitation Science, University of Central Florida, Orlando, FL 32816, USA; 4 College of Medicine, University of Central Florida, Orlando, FL 32827, USA; chitra.banarjee@ucf.edu; 5 Department of Statistics and Data Science, University of Central Florida, Orlando, FL 32816, USA; 6 Disability, Aging, and Technology Cluster, University of Central Florida, Orlando, FL 32816, USA; 7 Department of Robotics and Mechatronics Engineering, Daegu Gyeongbuk Institute of Science and Technology, Daegu 42988, Republic of Korea

**Keywords:** postural sway, ellipse area, sway speed variability, BTrackS

## Abstract

**Background/Objectives**: This study aimed to assess the impact of a physio-feedback exercise program (PEER) on the static balance of community-dwelling older adults. **Methods**: A clustered randomized controlled trial involving community-dwelling older adults (≥60 years of age) in the Central Florida area was conducted. Participants were randomized by research site into either (1) an 8-week exercise intervention program consisting of group-based and at-home exercises, along with a discussion with a researcher regarding their physiological health before and after the intervention period, or (2) a control group. Static balance outcomes included anterior–posterior root mean square (AP RMS), medial-lateral RMS (ML RMS), sway speed variability, and sway area measured using the Balance Tracking System (BTrackS) at baseline (T1), post-intervention (T2), one-month post-intervention (T3), and three months post-intervention (T4). **Results**: Among 373 community-dwelling older adults (mean age = 74.3 ± 7.1 years), a trend towards short-term improvement of sway area was observed for the intervention group, as seen through a small, marginally significant reduction in sway area at T2 (standardized β = −0.07; *p* = 0.050). However, the trend dissipated during post-intervention follow-up periods (T3 and T4). Sway speed variability significantly increased for the intervention group at T4 (standardized β = 0.10; *p* = 0.014). **Conclusions**: The PEER intervention may need to increase the total duration of the intervention, the frequency of the weekly exercise sessions, and the amount of standing stance exercises during the group-based and at-home exercise sessions to elicit improvements in static balance among older community-dwelling adults.

## 1. Introduction

Falls are common among older adults and often result in adverse consequences, such as the development of fear of falling, loss of mobility, hospitalizations, and increased medical costs [1,2,3]. It is projected that 100,000 older adult fatal falls will occur per year by 2030, along with an estimated $100 billion in medical costs [4]. To counteract this projection, various intervention programs have been developed and implemented among older adults with the goal of reducing fall risk and fall events, commonly using multifactorial approaches [5,6]. Such approaches include a culmination of computerized balance training, the implementation of group exercise classes, and biofeedback [7,8,9,10]. Intervention programs focus on improving key factors associated with fall risk and fall events, such as fear of falling, gait speed, and body composition [11,12]. Among these factors, postural balance during static standing is of great importance, as it is the foundation of overall balance control and directly determines an individual’s ability to maintain stability [13].

Postural balance during static standing can be defined as the ability to maintain one’s center of gravity within the weight support base during normal standing [14]. Postural balance can be evaluated through clinical assessments such as the Berg Balance Scale and the Functional Reach Test [15,16], as well as with automated methods such as force plate technology, which provide precise and detailed measurements [17,18]. Force plate technology is commonly used to measure static balance by tracking an individual’s center of pressure (COP) movement during a balance test, providing a detailed picture of their balance performance. Using the recorded COP movement, numerous metrics can be calculated to fully understand the static balance performance of an individual during a static balance test. Examples of static balance metrics that can be derived from force plate technology are sway area, sway speed, and ranges of sway in the anterior–posterior (AP) and medial-lateral (ML) directions. Some of these balance metrics have been found to have a relationship with fall risk. For example, previous research has found that individuals with higher values of sway area and sway distance exhibited a higher fall risk than those with lower values [19,20]. However, regarding fall risk, few studies have investigated other static balance metrics beyond sway area and sway distance. Previous research that has examined static balance metrics beyond sway distance and sway area for older adults have revealed specific movement tendencies that would not have been known if examining only sway distance and area [21,22]. Therefore, the inclusion of various metrics for the assessment of static balance performance is essential to fully understanding an individual’s ability to maintain stability.

An intervention program, named the Physio-feedback Exercise Program (PEER), has been previously developed that utilizes a combination of exercise (group and at-home), cognitive reframing, and performance feedback to reduce the fall risk and fall events among community-dwelling older adults [8]. This intervention was intended to decrease fall risk, improve dynamic balance, and increase physical activity of community-dwelling older adults, as these were defined as the primary outcomes of the trial. However, the intervention’s effect on static balance is of importance, as static balance can be considered as a prerequisite to dynamic balance. Preliminary work has found that the PEER intervention has potential benefits toward overall health including static balance performance [23,24,25], but the overall impact of this intervention on the static balance performance of community-dwelling older adults is currently unknown, as no previous studies that have examined the static balance of older adults who participated in the PEER intervention have examined static balance parameters beyond sway distance alone or included the effect of confounding variables. A clear understanding of the effect of this intervention on the static balance of community-dwelling older adults would aid clinicians and researchers in determining if this intervention is appropriate for the improvement of static balance performance for such population or if it needs to be modified to achieve such goals. For these reasons, an exploratory investigation beyond the primary outcomes of the trial was warranted.

This study aimed to perform an exploratory analysis to examine the effects of the PEER intervention on the static balance performance of community-dwelling older adults. Static balance performance for this study is represented by sway area, AP root mean square (RMS), ML RMS, and COP sway speed variability to gain a proper understanding of each individual’s static balance performance. Such metrics were selected to capture both directional variability and spatial distribution for each individual. Although sway distance is a simple and commonly used metric for representing static balance performance, it only presents the total movement of an individual’s COP, therefore lacking spatial and directional information. By considering overall sway area, magnitude of sway in both the AP and ML direction, and variability of sway speed, a more detailed assessment of each older adult’s postural control will be represented. To appropriately examine the effects of the PEER intervention on the four static balance metrics included in this study, the following variables were considered as covariates: age, BMI, sex, and race/ethnicity. It was hypothesized that the intervention group would exhibit a significant reduction in all static balance metrics post-intervention, symbolizing improved balance performance, whereas the control group would exhibit nonsignificant changes.

## 2. Materials and Methods

### 2.1. Study Design and Setting

This longitudinal study was conducted as part of an intent-to-treat, single-blinded, parallel, two-arm clustered randomized controlled trial federally funded by the National Institute on Minority Health and Health Disparities (R01MD018025) and pre-registered on ClinicalTrials.gov on 21 March 2023 (NCT05778604). The protocol for the trial and this study was approved by the University of Central Florida Institutional Review Board (STUDY00003206), carried out in accordance with the Declaration of Helsinki, and was previously published elsewhere [8]. This study took place in the Greater Orlando, Florida area between July 2023 and February 2025, and recruitment was accomplished using word-of-mouth, advertisement flyers, and partnerships with local community centers. Participants were included if they were at least 60 years of age, were not receiving treatment from any form of rehabilitation facility (e.g., acute, long-term), were low-income status based on the 2019 United States Census poverty thresholds relative to family size [26], and able to stand unassisted. Participants were excluded if they were unable to perform physical activity for any reason, hospitalized more than three times in the last year, or did not speak English or Spanish. All participants provided written informed consent prior to participation in the study. The trial has been reported in accordance with the CONSORT 2025 guidelines.

### 2.2. Sample Size Calculation

Utilizing information from previous studies, a sample size of at least 120 participants per arm was calculated to reach a statistical power of 80% to detect significant differences in repeated measures between the intervention and control group for the trial. A medium effect size (Cohen’s d = 0.25), intraclass correlation coefficient (ICC) of 0.8, and a significance level of 0.05 were used for the calculation of the sample size. Such information is outlined in the protocol [8].

### 2.3. Participants

A total of 404 community-dwelling older adults across 17 different sites in the Orlando area, consisting of older adult living centers and local community centers, were recruited for this study. Eligible participants were randomized by site into either the control or intervention group using the “randomizeR” function in RStudio (Version 4.3.3, Posit, Boston, MA, USA). The randomization process was conducted on a computer by the senior statistician on the research team alone. Only the statistician had access to the random allocation sequence. All participant data was deidentified, securely stored, and managed within a Research Electronic Data Capture (REDCap) database—a secure, web-based application designed to manage research data [27,28]. Four assessment periods took place for both the intervention and control group over the course of six months: at baseline (one week before the 8-week intervention period; T1), approximately one week after the 8-week intervention period (T2), one-month post-intervention (T3), and three months post-intervention (T4). During each assessment period, participants were asked to perform a static balance test using a commercial force plate system (Balance Tracking System; BTrackS) and complete a series of self-reported surveys. The research assistants performing the assessments were blinded to the participant’s assignment.

The primary outcomes of the trial itself were fall risk, dynamic balance, and physical activity levels, whereas the secondary outcomes were fall risk appraisal (comprising level of fear for falling and static balance performance) and negative self-perceptions of aging. In this exploratory analysis, the static balance of older adults was focused on to better understand the effects of the PEER intervention on static balance performance. Adverse events, represented by fall occurrence, were monitored by the research team through monthly check-ups conducted via telephone calls to the participants. Participants were instructed to contact a member of the research team if a fall occurred, if able.

#### 2.3.1. Intervention Group

Participants in the intervention group participated in an 8-week period of weekly group and at-home exercises, along with a discussion with a researcher regarding their static balance performance and fall risk pre-intervention and post-intervention. Group exercises occurred weekly for one hour for eight weeks with a trained physical instructor of similar background (i.e., age or culture) to the participants and were overseen by a certified exercise physiologist. Static stretching, low-load resistance exercises, and seated/standing balance exercises were performed during each group exercise session. Additionally, participants in the intervention group were asked to perform a minimum of one hour of at-home exercises weekly from a booklet that was developed by the research team and included similar exercises that were performed in the group exercise classes. The booklet comprised exercises categorized into seven categories: seated warm-ups, strengthening upper body (seated), strengthening upper body (standing), strengthening lower body (seated), strengthening lower body (standing), balance (standing), and balance (moving). Participants were given the opportunity to select any category of exercise to perform at home as long as a minimum of 60 min total was achieved during each week.

#### 2.3.2. Control Group

Participants in the control group were only given a variety of educational flyers from the Center for Disease Control (CDC) that presented information regarding mitigating fall risk in the home and in their daily lives. During the 8-week period for those in the control group, participants were instructed to live their lives normally.

### 2.4. Demographic Information

Demographic characteristics, such as age, general health, and sex, were collected for all included participants using self-reported surveys. Weight and height were objectively measured without shoes using a digital physician scale with a built-in stadiometer (Health-O-MeterTM, Model 402KL, McCook, IL, USA). Body mass index (BMI) was calculated using the determined height and weight and expressed as kg/m^2^.

### 2.5. Static Balance Assessment

The BTrackS (Balance Tracking Systems Inc., San Diego, CA, USA), a cost-effective force plate system, was used to assess static balance and record raw COP data. The BTrackS Balance and Fall Risk protocol was used as the static balance assessment for all participants and was administered through the BTrackS Assess Balance software (Version 7.5.5, Balance Tracking Systems Inc., San Diego, CA, USA). The protocol consisted of four 20 s static standing trials. The first trial served as a practice trial, whereas the following three trials were used for the calculation of static balance metrics. For each trial, participants were instructed to stand on the force plate with feet shoulder width apart with a toe-out angle of zero degrees, hands on hips, head facing forward, and eyes closed. A member of the research team placed their hand near the participant’s back and continuously watched the participant during each trial to ensure safety of the participant. These procedures follow the Balance and Fall Risk protocol. Raw COP values from the BTrackS were obtained every 40 milliseconds by sampling the four load cells within the force plate. The COP values were then passed through a 2nd order, low-pass Butterworth filter with a 4 Hz cut-off frequency. Raw COP movement data in the x and y directions were utilized to calculate the following static balance metrics: sway area, AP RMS, ML RMS, and sway speed variability. Sway area was defined as the area of the ellipse that covers 95% of the COP path, AP RMS as the average magnitude of sway in the AP direction, ML RMS as the average magnitude of sway in the ML direction, and sway speed variability as the standard deviation of the sway velocity. Each metric was calculated separately for each of the three trials and then averaged across the trials. Due to the multiple trials within each balance assessment, data was controlled by averaging the results from each of the three balance trials at each timepoint in the study to mitigate the effects of outliers. Additionally, each researcher administrating the balance assessment was trained to give a specific set of instructions that closely followed the instructions provided by the BTrackS Balance and Fall Risk protocol and the BTrackS device, along with its accompanying software, was held consistently for each research site throughout the duration of the study. The BTrackS, as well as the Balance and Fall Risk protocol, has been used in previous research with older adults and has shown to be reliable and accurate for assessing fall risk [29].

### 2.6. Statistical Analysis

All statistical analysis was performed using RStudio (Version 4.3.3, Posit, Boston, MA, USA). Anderson-darling tests found that the four static balance variables, as well as age and BMI, exhibited nonnormal distributions and therefore, continuous demographic data for each group at baseline was compared through Mann–Whitney U tests. Categorical demographic data was compared through Fisher’s exact test due to certain categories having a very small sample size (<5). Similarly, fall occurrences between groups were compared through Fisher’s exact test. Specifically, fall frequency (one fall vs. multiple) and proportion of fallers between groups was compared. A logarithmic transformation was applied to all balance variables to approximate a normal distribution for analysis. To account for the cluster-randomized design for this study, linear mixed effects models were fitted to the four continuous outcomes (AP RMS, ML RMS, sway speed variability, and sway area) to assess short- (T1–T2) and medium- term (T1–T4) effects through the “lme” function in the “lme4” package in RStudio [30]. Linear mixed effects models were chosen due to their robustness to violations in normality [31]. To account for any confounding effects, the following variables were considered as covariates for analysis: age, BMI, sex, and race/ethnicity. Each model consisted of random effects for the subject IDs assigned to each participant and the research sites that were included in this study, as well as fixed effects for group (intervention vs. control), timepoint (T1–T4), age, BMI, sex, race/ethnicity, and an interaction between group and timepoint. The models were optimized using the “bobyqa” optimizer and utilized restricted maximum likelihood (REML) estimation to account for the random effects. ICCs were utilized to determine the amount of variance explained by each of the random effects and R^2^ to assess the overall performance of each model. Observed power for the interaction effect between timepoint and group was estimated using simulation-based methods for each static balance metric using the “simr” package in RStudio. Participants with missing data at various timepoints were included in the analysis, as linear mixed effects models can accommodate missing data [32,33]. Effect sizes were quantified using R^2^ values for each model and standardized fixed-effect coefficients for each predictor determined using the “standardize_parameters” function from the “effectsize” package. Standardized fixed-effect coefficients impose a common scale across predictors, allowing for the strength of an association to be more easily understood. Data are presented as mean ± standard deviation unless indicated otherwise, and the threshold for significance was set at *p* < 0.05.

## 3. Results

**Figure 1:**
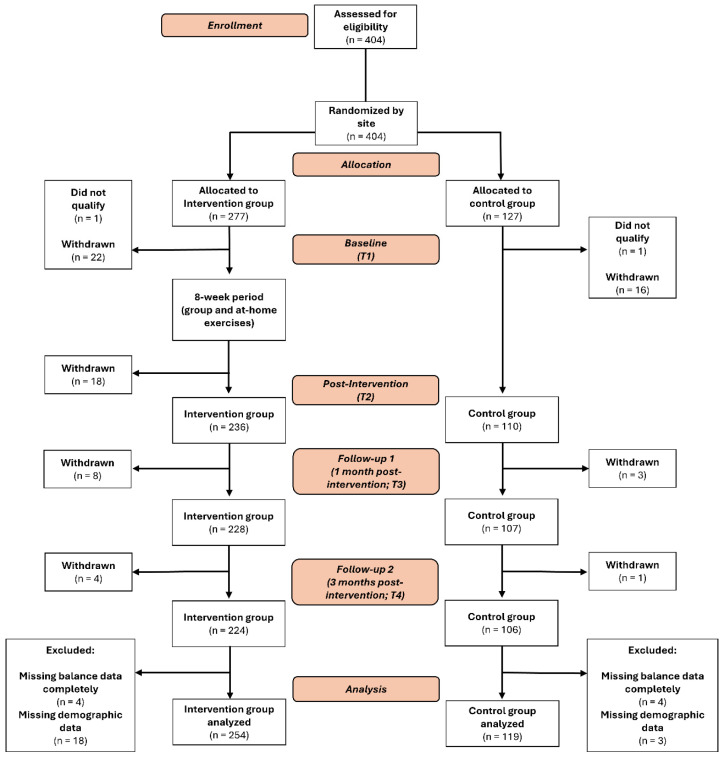
Flow of participants throughout the study.

After screening for inclusion and exclusion criteria, a total of 373 community-dwelling older adults were included in this study (mean age = 74.3 ± 7.1 years). Participants who did not have static balance data for any of the four timepoints (T1–T4) or had missing demographic data were excluded. Adherence to the group-based exercise sessions was 78.0 ± 26.7%, whereas adherence to the at-home exercises was 69.2 ± 31.8%. The distribution of participants throughout this study has been presented in Figure 1. Baseline characteristics for all participants have been presented in Table 1.

All balance metrics, age, sex, self-reported health, financial status, and living status did not significantly differ between the control and intervention groups at T1. BMI and race/ethnicity significantly differed between groups T1. The intervention group had a significantly lower BMI when compared to the control group. Additionally, the intervention group included a higher percentage of White participants and a lower percentage of African American participants. Raw mean and standard deviations for all balance metrics for each group from T1 through T4 are presented in Table 2.

### 3.1. Linear Mixed Effects Models for All Static Balance Metrics

Table 3 presents the results from the linear mixed effects models fitted for AP RMS, ML RMS, sway speed variability, and sway area. Table 4 expands on Table 3 by showing the same four models and the associated predictors but with their respective standardized fixed-effect coefficients and 95% confidence intervals. Age and BMI were significantly associated with all balance metrics (*p* < 0.05). Sex and race/ethnicity, specifically Asian, were significantly associated with sway speed variability. AP RMS, ML RMS, and sway area did not significantly change across the four time points. However, a small, marginally significant reduction in sway area for the intervention group relative to the control group was observed at T2 (Std β = −0.07; *p* = 0.050). Additionally, a significant interaction effect was observed in sway speed variability for the intervention group at T4, finding that the intervention group’s sway speed variability slightly increased from T1 to T4 relative to the control group (Std β = 0.10; *p* = 0.014).

**Figure 2:**
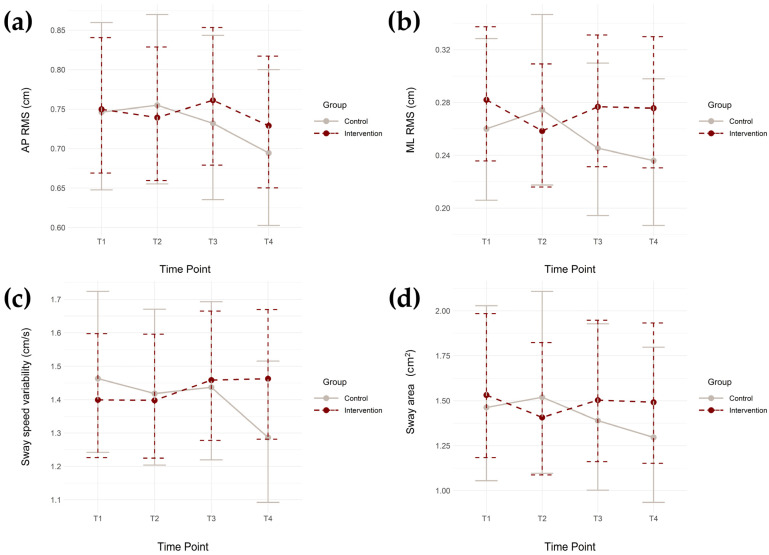
Estimated marginal means and their associated 95% confidence intervals from each linear mixed effects model compared across all timepoints. (**a**) anterior–posterior root mean square (AP RMS), (**b**) medial-lateral root mean square (ML RMS), (**c**) sway speed variability, and (**d**) sway area.

The estimated marginal means from each linear mixed effects model at the four timepoints, along with their 95% confidence intervals (CIs), are presented in Figure 2.

### 3.2. Number of Falls Among Groups

Out of the 373 older adults included, 55 experienced at least one fall during their participation in the study. A total of 34 older adults in the intervention group experienced at least one fall (30 experienced only one fall), whereas a total of 21 experienced at least one fall in the control group (16 experienced only one fall). No significant differences were found between groups in either the proportion of participants who experienced a fall or in the number of falls among those who fell (*p* = 0.279 for both tests). The number of fall occurrences is shown in Table 5.

## 4. Discussion

The purpose of this study was to examine short- and medium-term effects on the static balance performance of community-dwelling older adults who participated in the 8-week PEER intervention compared to those who were a control group, while considering age, BMI, sex, and race/ethnicity. When comparing group characteristics at baseline, the intervention group had a significantly lower BMI than that of the control group, as well as a greater percentage of White participants and lower percentage of African American participants. Our original hypothesis was not supported, as no significant improvements were found for any of the four static balance metrics at any timepoint post-intervention. However, a small, marginally significant improvement in sway area was found post-intervention (T2) for the intervention group (Std β = −0.07; *p* = 0.050), indicating a possible trend towards reduction in sway area (Table 3 and Table 4, and Figure 2d). The trend dissipated in the follow-up periods (T3 and T4). Regarding sway speed variability, there was a significant interaction effect between group and timepoint at T4 (Std β = 0.10; *p* = 0.014), indicating a small increase in sway speed variability in the intervention group at T4 compared to baseline (Table 3 and Table 4, and Figure 2c). Additionally, the PEER intervention did not significantly impact fall occurrence when compared to the control group. The results suggest that the current physio-feedback program may not significantly improve specific directional sway tendencies or variability of sway speed. However, it may provide short-term improvements in static balance performance, as seen through the marginally significant reduction in sway area immediately after the intervention period. These improvements do not appear to persist once the in-person group exercise classes and accountability for at-home exercises (via exercise logs) ceased, suggesting that older adults may not benefit from self-guided interventions (such as the T2–T4 time period) for improving static balance performance. Alterations to the intervention should be considered to elicit such improvements.

COP sway range was initially considered as a metric for this study, as it is an indicator of COP movement in the AP and ML directions. However, it is limited by its sensitivity to brief, extreme movements. For example, an individual may maintain stability within a narrow range in the AP direction for most of a balance test, but once a single sudden movement occurs, the sway range in the AP direction increases drastically, resulting in an outlier that might not accurately represent an individual’s true range of sway in the AP direction. RMS provides the average movement of postural sway and has been previously used as a measure of static balance performance as it is less sensitive to extremes/outliers, hence why it was chosen over sway range for this study [13]. Although the sway range metric was not analyzed due to its sensitivity, it is possible that the intervention group may have experienced reductions in sway in one (or both) directions and such reductions would justify the improvements in sway area for the intervention group, despite the nonsignificant improvements in the AP and ML RMS metrics. The models for the RMS variables in this study exhibited the lowest conditional R^2^ values out of the static balance metrics considered, with ML RMS having the lowest out of the two (0.52). Additionally, the observed statistical power for the models fitted for both RMS metrics was low to moderate, with the AP RMS model being lower than that of the ML RMS model (22% and 68%, respectively). The low to moderate statistical power, along with other factors that possibly may have confounded the ML and AP RMS metrics, may have contributed to the nonsignificant changes found in the intervention group.

Sway speed variability significantly increased for the intervention group during the follow-up period post-intervention (Table 3 and Table 4, and Figure 2c). While the other static balance metrics did not exhibit this trend, sway speed variability can be defined as the variation in speed at which an individual’s COP moves during static standing. It has been previously found that increased sway speed has associations with neurological conditions or events such as a stroke, Parkinson’s disease, and diabetic peripheral neuropathy [13,34,35]. It is possible that individuals in the intervention group may have experienced the development or worsening (if previously diagnosed) of such conditions during the post-intervention follow-up sessions (T3 and T4). Other adverse events, both intrinsic and extrinsic (e.g., loss of motivation, development of depression, difficulty accessing gym equipment), may have also contributed to this result. However, the variation in an individual’s sway speed does not necessarily signify poor overall balance performance. Velocity-based metrics regarding balance performance can reveal control strategies and corrective responses. Increased sway speed variability could denote increased movement or faster corrective responses to COP shifts. An increase in corrective response velocity as a result of a shift in COP may signify improved balance performance. However, such improvements may contribute more towards dynamic balance assessments such as the Timed-Up-and-Go or Short Physical Performance Battery (SPPB), due to the larger amounts of weight shifts occurring during dynamic movements (e.g., walking). Overall, an investigation of additional static balance metrics relating to sway speed is required to fully determine if the increase in sway speed variability benefits static balance performance.

Previous studies have examined the effects of an exercise intervention on the static balance of older adults and have found significant improvements in static balance performance. One study that implemented a 9-week group-based exercise intervention comparing the effects of strength training exercises and neuromuscular electric stimulation found that significant improvements in sway velocity and sway in both the ML and AP directions, as found using a force plate, occurred in both intervention groups when compared to the control group [36]. A group-based intervention comprising stretching and seated/standing balance exercises was previously implemented for a duration of 12 weeks and found significant improvements (reduction) in COP deviation in the ML and AP directions, as found through force plate measurements. The effects of the intervention persisted as long as 12 months post-intervention [37]. However, such intervention was implemented on older adults who had already experienced a fall along with a hip fracture, and these conditions may have been severe enough to elicit a drastic improvement in static balance performance. Another study implemented biweekly group-based exercise sessions including tai chi-derived movements and leg strengthening exercises using elastic bands for 12 weeks along with home-based exercises and found significant improvements in static balance, as determined through one-legged stance tests for each leg for both eyes open and closed conditions [38]. Additionally, a study implementing group-based sensory-based static balance training for a duration of 40 min three times a week for 12 weeks on older adults found significant improvements in static balance performance, as defined by the One Leg Stand Test, immediately after the intervention period, with the improvement maintaining as long as 12-weeks post-intervention [39]. The intervention itself only included standing balance exercises, which may have contributed to medium-term improvements in static balance due to the intensive emphasis on static standing balance performance alone.

In contrast, a few studies found no significant improvements in static balance for older adults despite the implementation of an exercise intervention. A study that implemented tai-chi-inspired exercises, along with dancing and step-based exercises, performed three times a week for 30 to 40 min for 16 weeks did not observe significant improvements in balance, as described by the score of the balance portion of the SPPB [40]. While the duration of the intervention was comparable to the previously mentioned studies, this intervention was self-guided and performed individually in one’s home without supervision, which may have contributed to the nonsignificant improvements. Another study implemented a 12-week group-based intervention comprising two sessions per week that included lower limb resistance training exercises in seated and standing positions. Nonsignificant changes in static balance were found, as assessed through a tandem stand test and one-leg stand test [41].

Overall, the efficacy of an exercise intervention to improve the static balance of older adults is dependent on numerous factors, namely the health condition of the group being trained, the modality to which static balance is assessed, the type of exercise being performed, whether the exercise is self-guided or guided by a professional, and the duration of the overall intervention. Based off previous work, exercises that specifically strengthen the legs, namely the muscles that contribute to AP and ML stabilization (e.g., dorsiflexors and plantar flexors of the ankle, hip abductors and adductors), may be the most effective exercises towards improving static balance performance, as leg weakness is a common and important fall risk factor [42,43,44]. Additionally, balance training exercises (exercises that test the body’s ability to maintain its center of gravity over its base of support [45]) have been recommended to reduce the risk of falls in older adults [42,46]. A review of balance training methodologies found that the most effective balance training interventions to improve the static balance performance of healthy older adults consist of a training period lasting 11–12 weeks with a total of 36–40 training sessions each 31–45 min in duration and occurring three times a week [47]. The nonsignificant improvements in static balance performance found in the current study could be attributed to the overall duration of the PEER intervention, as well as the frequency of the exercise classes. Although the exercises in the PEER intervention engaged the muscles relating to AP and ML stabilization, the intervention was both shorter in duration and less frequent than what has been shown to be most effective for balance training interventions. Additionally, although the weekly group-based exercise sessions included exercises that targeted static balance performance specifically, the at-home component gave participants the freedom to choose their exercise to meet the 60-min goal. It is possible that the focus turned away from balance-improving exercises specifically during the at-home portion of the intervention. The findings from this exploratory study indicate to clinicians and researchers that the PEER intervention in its current form may not be suitable for improving the static balance performance of community-dwelling older adults, as represented by the small changes in static balance performance. Based off previous work that found an improvement in static balance performance in older adults, clinicians and researchers may need to increase the total duration of the PEER intervention, as well as frequency of the exercise classes within the intervention, and specify the type of exercise being performed at each individual’s home to significantly improve the static balance performance of community-dwelling older adults. Such alterations may need to be made before the intervention can be implemented on a larger scale and associated with improvements in static balance performance among older adults.

### Limitations

While this study implemented a strong design, as evidenced by its cluster-randomized approach and the selection of robust procedures for statistical analysis, certain limitations remain. Ideally, the BTrackS Balance and Fall Risk protocol should be conducted in an area that has minimal noise or distractions. Due to conditions at certain research sites included in this study, surrounding noise and distractions (e.g., other events occurring in the same room) could not be completely controlled or mitigated. Although the research team made every effort to minimize these factors, complete mitigation was not always possible. As a result, the performance of some older adult participants may have been affected. However, the results from this study found that the site random effect included in the statistical models for all static balance metrics did not contribute to a significant amount of variation (*p* > 0.05). Additionally, although the recruitment and screening process was open to all older adults at each of the research sites included, a greater number of females expressed interest in participating in the study than males. As a result, the overall sample size is dominantly female (88%). This dominance may limit the generalizability of the results presented.

The freedom given to the participants of the PEER intervention regarding at-home exercises exists as a potential source of bias. Although the participants enjoyed the freedom of selecting their own preference of exercise within the seven categories given and the type of exercise, as well as the frequency, was self-reported by each participant, the exercise intensity remains unknown. Additionally, self-reported measures may have been influenced by the social desirability to adhere to the study’s requirements. This form of bias may have caused participants to overreport their weekly at-home activities.

The sample size needed to achieve a statistical power of 80% to appropriately detect differences between the intervention and control group over repeated measures was at least 120 participants per arm. While the number of participants collectively recruited and randomized to each group in this study at baseline surpassed the amount needed to achieve a statistical power of 80%, a reduction in sample sizes for this analysis occurred due to missing or unlabeled balance assessment data. For numerous participants, the static balance test at each assessment period was conducted more than once for various reasons and was left unlabeled, leaving their true performance unknown at certain time points. Although this reduction occurred, the observed power for the linear mixed effect models fitted for sway speed variability and sway area surpassed the expected 80% (83% for both models). However, the models for the RMS metrics exhibited observed powers below 80% (AP RMS = 22%, ML RMS = 68%) and therefore, should be interpreted with caution. Future research should aim to accurately label balance data when multiple balance assessments are taken for any reason to avoid reducing overall sample data size, as well as ensure that all balance assessments are performed in areas that have minimal to no distractions.

## 5. Conclusions

This study found a trend towards short-term improvements in sway area, as found through a small, marginally significant reduction in the intervention group post-intervention. A significant, but small, increase in sway speed variability was found in the intervention group post-intervention, possibly revealing an improvement in corrective response velocity to shifts in COP. However, due to the nonsignificant improvements in other static balance metrics, such results could contribute more towards improved dynamic balance performance. Overall, the changes were small and therefore should be interpreted with caution. The results of this study warrant future research regarding the effect of the PEER intervention on static balance performance. The absence of short- and medium-term improvements to static balance performance may be attributed to the short overall duration of the intervention, the low frequency of exercise classes, and a possible shift in participant’s home-based exercises away from balance-focused exercises. Based on the findings of previous studies, the PEER intervention may need to increase the total duration of the intervention, increase the number of weekly exercise sessions, and place more emphasis on standing stance exercises during the group-based and at-home exercise sessions to elicit improvements in the static balance performance of community-dwelling older adults.

## Figures and Tables

**Table 1:** Participant characteristics at baseline.

		Baseline			
Demographics	Category	Total (*n* = 373)	Intervention (*n* = 254)	Control (*n* = 119)	*p*
Age (years)		74.3 ± 7.1	74.3 ± 6.9	74.3 ± 7.6	0.788
BMI (kg/m^2^)		30.2 ± 6.2	29.7 ± 5.8	31.4 ± 6.9	0.022 ^1^
Sex	Female	327	225	102	0.538
	Male	46	29	17	
Race/ethnicity	African American	176	104	72	0.005 ^1^
	Asian	22	15	7	
	Hispanic	93	69	24	
	White	75	61	14	
	Other	7	5	2	
Self-reported health	Excellent	22	13	9	0.651
	Very Good	113	81	32	
	Good	172	117	55	
	Fair	57	36	21	
	Poor	9	7	2	
Financial status	Much more than adequate	11	8	3	0.772
	More than adequate	68	48	20	
	Just enough	217	149	68	
	Less than adequate	53	32	21	
	Much less than adequate	24	17	7	
Living status	Alone	198	138	60	0.734
	Partner/ Spouse	87	60	27	
	Family/friend	75	47	28	
	Other	13	9	4	
AP RMS (cm)		0.84 ± 0.38	0.83 ± 0.37	0.86 ± 0.42	0.494
ML RMS (cm)		0.32 ± 0.20	0.31 ± 0.21	0.33 ± 0.19	0.263
Sway speed variability (cm/s)		1.56 ± 0.80	1.52 ± 0.75	1.66 ± 0.91	0.233
Sway area (cm^2^)		2.19 ± 2.43	2.06 ± 2.13	2.50 ± 2.97	0.326

^1^ Statistically significant value (*p* < 0.05). Note: AP RMS = anterior–posterior root mean square; BMI = body mass index; ML RMS = medial-lateral root mean square; *n* = sample size.

**Table 2:** Raw mean and standard deviation of static balance metrics from T1–T4.

Variable	T1		T2		T3		T4	
	Intervention	Control	Intervention	Control	Intervention	Control	Intervention	Control
Participants with available data	247	111	212	93	196	85	193	83
AP RMS (cm)	0.83 ± 0.37	0.86 ± 0.42	0.82 ± 0.36	0.89 ± 0.44	0.82 ± 0.33	0.88 ± 0.49	0.80 ± 0.34	0.79 ± 0.33
ML RMS (cm)	0.31 ± 0.21	0.33 ± 0.19	0.31 ± 0.23	0.35 ± 0.24	0.31 ± 0.21	0.35 ± 0.36	0.30 ± 0.18	0.27 ± 0.16
Sway speed variability (cm/s)	1.52 ± 0.75	1.66 ± 0.91	1.53 ± 0.76	1.68 ± 1.05	1.51 ± 0.66	1.73 ± 1.12	1.54 ± 0.72	1.41 ± 0.60
Sway area (cm^2^)	2.06 ± 2.13	2.50 ± 2.97	1.87 ± 1.95	2.86 ± 4.34	1.89 ± 1.70	3.53 ± 9.13	1.88 ± 1.56	1.78 ± 1.50

Note: AP RMS = anterior–posterior root mean square; ML RMS = medial-lateral root mean square.

**Table 3:** Results of linear mixed effects models fitted for all static balance metrics.

Predictors	AP RMS (Log-Transformed)			ML RMS (Log-Transformed)			Sway Speed Variability (Log-Transformed)			Sway Area (Log-Transformed)		
	Estimate	95% CI	*p*	Estimate	95% CI	*p*	Estimate	95% CI	*p*	Estimate	95% CI	*p*
(Intercept)	−1.17	(−1.66, −0.67)	<0.001 ^1^	−2.71	(−3.40, −2.03)	<0.001 ^1^	−0.91	(−1.48, −0.34)	0.002 ^1^	−2.07	(−3.12, −1.02)	<0.001 ^1^
Group (intervention vs. control)	−0.02	(−0.12, 0.08)	0.667	−0.03	(−0.20, 0.14)	0.729	−0.04	(−0.17, 0.09)	0.531	−0.07	(−0.30, 0.15)	0.522
Time (T2)	0.03	(−0.05, 0.11)	0.449	0.06	(−0.05, 0.18)	0.277	0.01	(−0.08, 0.09)	0.837	0.09	(−0.03, 0.20)	0.140
Time (T3)	0.00	(−0.08, 0.08)	0.975	−0.06	(−0.17, 0.06)	0.355	0.02	(−0.07, 0.10)	0.727	−0.01	(−0.13, 0.11)	0.833
Time (T4)	−0.05	(−0.14, 0.03)	0.206	−0.10	(−0.22, 0.02)	0.089	−0.10	(−0.19, −0.01)	0.028 ^1^	−0.10	(−0.21, 0.02)	0.118
Age	0.01	(0.00, 0.01)	0.020 ^1^	0.02	(0.01, 0.02)	<0.001 ^1^	0.01	(0.00, 0.02)	0.002 ^1^	0.02	(0.01, 0.04)	<0.001 ^1^
BMI	0.01	(0.00, 0.02)	<0.001 ^1^	0.01	(0.00, 0.02)	<0.006 ^1^	0.01	(0.01, 0.02)	<0.001 ^1^	0.02	(0.01, 0.03)	0.001 ^1^
Sex (female vs. male) ^2^	0.08	(−0.02, 0.19)	0.126	0.06	(−0.08, 0.21)	0.398	0.13	(0.01, 0.26)	0.034 ^1^	0.20	(−0.02, 0.43)	0.076
Race/ethnicity (African American) ^3^	0.13	(−0.13, 0.39)	0.336	−0.16	(−0.52, 0.20)	0.383	0.17	(−0.13, 0.47)	0.274	−0.03	(−0.58, 0.51)	0.902
Race/ethnicity (Asian) ^3^	0.26	(−0.03, 0.55)	0.085	−0.02	(−0.43, 0.38)	0.910	0.36	(0.02, 0.69)	0.040 ^1^	0.22	(−0.40, 0.84)	0.486
Race/ethnicity (Hispanic) ^3^	0.10	(−0.16, 0.36)	0.450	−0.16	(−0.53, 0.21)	0.388	0.13	(−0.18, 0.43)	0.405	−0.09	(−0.65, 0.47)	0.754
Race/ethnicity (White) ^3^	0.25	(−0.02, 0.51)	0.069	−0.09	(−0.46, 0.28)	0.631	0.29	(−0.01, 0.60)	0.061	0.17	(−0.39, 0.73)	0.550
Group * Time (T2)	−0.04	(−0.14, 0.05)	0.389	−0.09	(−0.23, 0.05)	0.196	−0.01	(−0.11, 0.09)	0.869	−0.14	(−0.28, 0.00)	0.050
Group * Time (T3)	−0.01	(−0.10, 0.09)	0.915	0.03	(−0.11, 0.18)	0.629	0.00	(−0.11, 0.10)	0.938	−0.02	(−0.16, 0.12)	0.792
Group * Time (T4)	0.02	(−0.08, 0.12)	0.678	0.10	(−0.04, 0.24)	0.172	0.13	(0.03, 0.24)	0.014 ^1^	0.08	(−0.07, 0.22)	0.293
	**AP RMS** **(Log-Transformed)**			**ML RMS** **(Log-Transformed)**			**Sway Speed Variability** **(Log-Transformed)**			**Sway Area** **(Log-Transformed)**		
**Random effects**												
Residual variance (σ^2^)	0.08			0.16			0.09			0.16		
Subject variance (τ_00 studyID_)	0.08		<0.001 ^1^	0.15		<0.001 ^1^	0.11		<0.001 ^1^	0.44		<0.001 ^1^
Site variance (τ_00 site_)	0.00		1.000	0.01		0.142	0.00		0.340	0.01		0.557
ICC_subjectID_	0.51			0.47			0.56			0.72		
ICC_site_	0.01			0.03			0.02			0.02		
N_studyID_	373			373			373			373		
N_site_	17			17			17			17		
Observations	1220			1220			1220			1220		
Marginal R^2^	0.06			0.05			0.08			0.09		
Conditional R^2^	0.54			0.52			0.61			0.76		

^1^ Statistically significant value (*p* < 0.05). ^2^ Reference was “Female” category. ^3^ Reference was “Other” category. Note: AP RMS = anterior-posterior root mean square; ML RMS = medial-lateral root mean square; * displays interaction effect.

**Table 4:** Standardized fixed-effect coefficients and their associated 95% confidence intervals for each of the fitted linear mixed effects models.

Predictors	AP RMS (Log-Transformed)			ML RMS (Log-Transformed)			Sway Speed Variability (Log-Transformed)			Sway Area (Log-Transformed)		
	Std β	95% CI	*p*	Std β	95% CI	*p*	Std β	95% CI	*p*	Std β	95% CI	*p*
(Intercept)	0.79	(0.57, 1.01)	<0.001 ^1^	0.78	(0.54, 1.02)	<0.001 ^1^	0.70	(0.46, 0.94)	0.002 ^1^	0.43	(0.21, 0.65)	<0.001 ^1^
Group (intervention vs. control)	−0.02	(−0.10, 0.07)	0.667	−0.02	(−0.13, 0.08)	0.729	−0.04	(−0.14, 0.06)	0.531	−0.04	(−0.13, 0.05)	0.522
Time (T2)	0.03	(−0.04, 0.10)	0.449	0.05	(−0.03, 0.12)	0.277	0.00	(−0.06, 0.07)	0.837	0.04	(−0.01, 0.09)	0.140
Time (T3)	0.00	(−0.07, 0.07)	0.975	−0.02	(−0.10, 0.05)	0.355	0.01	(−0.05, 0.08)	0.727	0.02	(−0.03, 0.07)	0.833
Time (T4)	−0.05	(−0.12, 0.02)	0.206	−0.07	(−0.14, 0.01)	0.089	−0.08	(−0.15, −0.01)	0.028 ^1^	−0.04	(−0.09, 0.01)	0.118
Age	0.04	(0.01, 0.07)	0.020 ^1^	0.07	(0.04, 0.10)	<0.001 ^1^	0.05	(0.02, 0.08)	0.002 ^1^	0.07	(0.04, 0.10)	<0.001 ^1^
BMI	0.05	(0.02, 0.09)	<0.001 ^1^	0.04	(0.01, 0.08)	<0.006 ^1^	0.06	(0.02, 0.09)	<0.001 ^1^	0.05	(0.01, 0.08)	0.001 ^1^
Sex (female vs. male) ^2^	0.07	(−0.02, 0.16)	0.126	0.04	(−0.05, 0.13)	0.398	0.11	(0.02, 0.21)	0.034 ^1^	0.09	(0.00, 0.18)	0.076
Race/ethnicity (African American) ^3^	0.10	(−0.12, 0.32)	0.336	−0.08	(−0.31, 0.14)	0.383	0.12	(−0.11, 0.35)	0.274	−0.01	(−0.23, 0.21)	0.902
Race/ethnicity (Asian) ^3^	0.21	(−0.04, 0.45)	0.085	0.00	(−0.25, 0.26)	0.910	0.27	(0.01, 0.54)	0.040 ^1^	0.10	(−0.15, 0.34)	0.486
Race/ethnicity (Hispanic) ^3^	0.08	(−0.14, 0.30)	0.450	−0.08	(−0.31, 0.15)	0.388	0.09	(−0.14, 0.33)	0.405	−0.04	(−0.26, 0.18)	0.754
Race/ethnicity (White) ^3^	0.20	(−0.02, 0.42)	0.069	−0.04	(−0.27, 0.19)	0.631	0.22	(−0.02, 0.46)	0.061	0.07	(−0.15, 0.30)	0.550
Group * Time (T2)	−0.04	(−0.12, 0.04)	0.389	−0.06	(−0.15, 0.03)	0.196	0.00	(−0.08, 0.07)	0.869	−0.07	(−0.13, −0.01)	0.050
Group * Time (T3)	0.00	(−0.09, 0.08)	0.915	0.01	(−0.08, 0.10)	0.629	0.00	(−0.09, 0.07)	0.938	−0.04	(−0.11, 0.02)	0.792
Group * Time (T4)	0.02	(−0.06, 0.10)	0.678	0.06	(−0.03, 0.15)	0.172	0.10	(0.02, 0.18)	0.014 ^1^	0.02	(−0.04, 0.09)	0.293

^1^ Statistically significant value (*p* < 0.05). ^2^ Reference was “Female” category. ^3^ Reference was “Other” category. Note: AP RMS = anterior–posterior root mean square; ML RMS = medial-lateral root mean square; Std β = standardized fixed-effect coefficient; * displays interaction effect.

**Table 5:** Participants that experienced a fall throughout the study.

Group	Experienced at Least One Fall	Experienced One Fall	Experienced More Than One Fall	Experienced No Fall
Control	21	16	5	98
Intervention	34	30	4	220

## Data Availability

The deidentified data for this study, as well as the statistical processing code, is publicly available at https://osf.io/8763w/overview (created 25 October 2025).

## Abbreviations

The following abbreviations are used in this manuscript:

AP	Anterior–posterior
COP	Center of pressure
ML	Medial-lateral
PEER	Physio-feedback Exercise Program
RMS	Root mean square
